# Supplementary material for: Ultrasensitive single-step CRISPR detection of monkeypox virus in minutes with a vest-pocket diagnostic device
Source: Nat Commun. 2024 Apr 16;15:3279. doi: 10.1038/s41467-024-47518-8 (PMC11021474; doi:10.1038/s41467-024-47518-8)
Supplement: Supplementary file 1 — Supplementary Information [file 41467_2024_47518_MOESM1_ESM.pdf]

Supplementary Materials for

Ultrasensitive single-step CRISPR detection of monkeypox virus in minutes with a vest-pocket diagnostic device

Yunxiang Wang[1,†], Hong Chen[1,†], Kai Lin[2,†], Yongjun Han[1,†], Zhixia Gu[3], Hongjuan Wei[1], Kai Mu[1], Dongfeng Wang[1], Liyan Liu[1], Ronghua Jin[3,*], Rui Song[3,*], Zhen Rong[1,*] & Shengqi Wang[1,*]

[1] Bioinformatics Center of AMMS, Beijing 100850, China.

[2] Department of Clinical Laboratory, Air Force Medical Center, Air Force Medical University, Beijing 100142, China.

[3] Beijing Ditan Hospital, Capital Medical University, Beijing 100015, China.

[†]Y.-X. Wang, H. Chen, K. Lin, and Y.-J. Han contributed equally to this work.

Corresponding Author

*Shengqi Wang, E-mail: sqwang@bmi.ac.cn.

*Zhen Rong, E-mail: rongzhen0525@sina.com.

*Rui Song, E-mail: songruii@hotmail.com.

*Ronghua Jin, E-mail: ronghuajin@ccmu.edu.cn.

This PDF file includes:
Supplementary Fig. 1 to 14
Supplementary Table 1 to 7

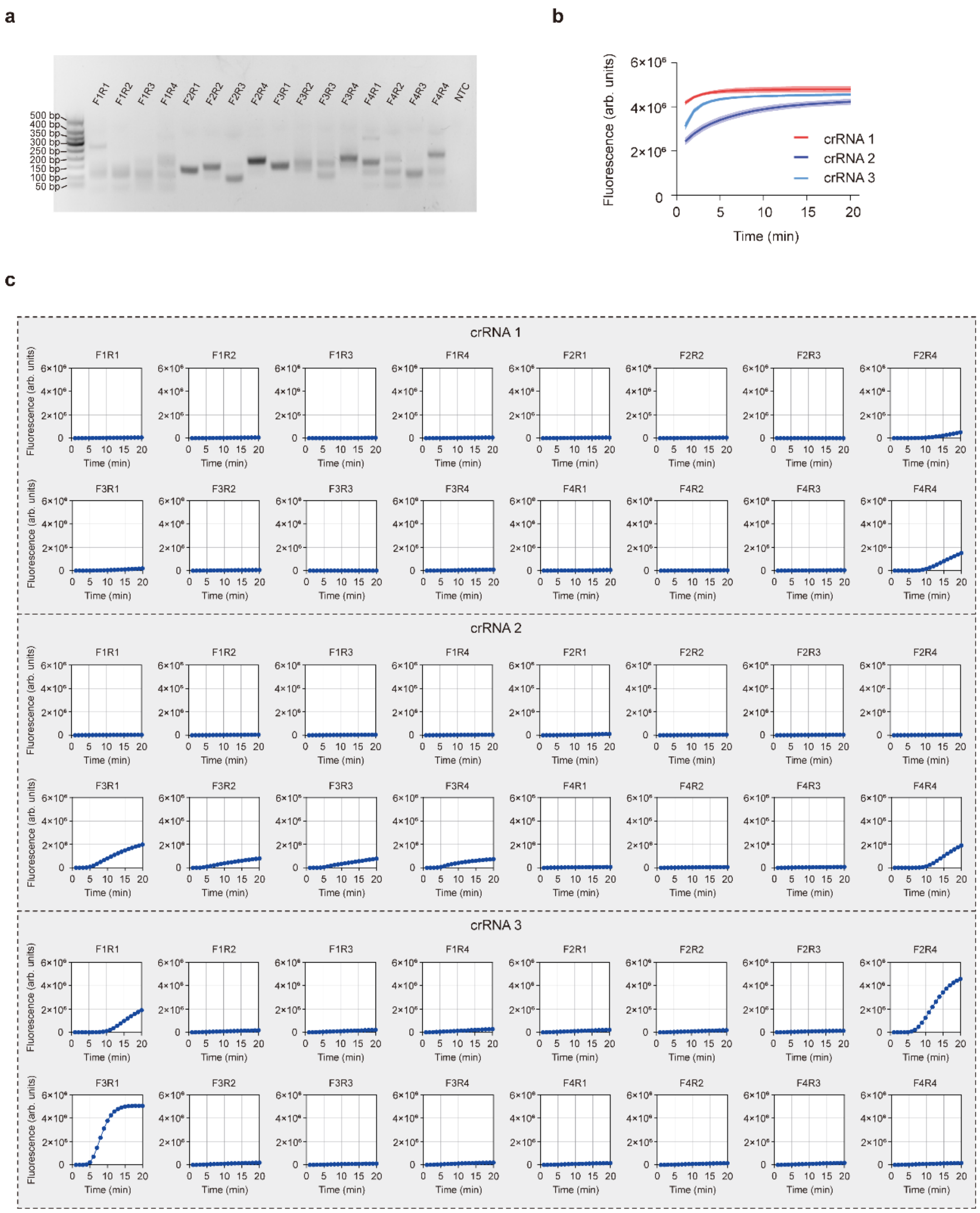

Supplementary Fig. 1. Primers and crRNAs screening. a, The gel image of 16 pairs of RPA primers screening via 3% agarose gel electrophoresis. **b**, Real-time fluorescence curves of Cas13a reaction for 3 crRNA screening. **c**, Real-time fluorescence curves of RPA primers and Cas13a crRNA simultaneous screening were tested by plate reader using the single-step RPA-CRISPR/Cas13a reaction for 20 minutes. Target: monkeypox virus F3L gene of 10 copies/μL.

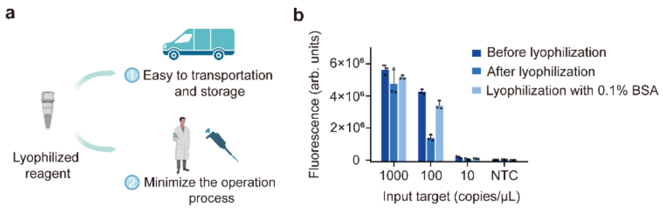

Supplementary Fig. 2. Reagent lyophilization. **a**, The lyophilized regent was convenient for transportation, storage, and operation. **b**, The end-point fluorescence intensity of the 10-minute single-step CRISPR reaction using reagents before lyophilization, after lyophilization, and lyophilization with 0.1% BSA with different concentrations of monkeypox virus DNA (1000, 100, 10, and 0 copies). Mean ± s.d. for 3 technical replicates for **b**.

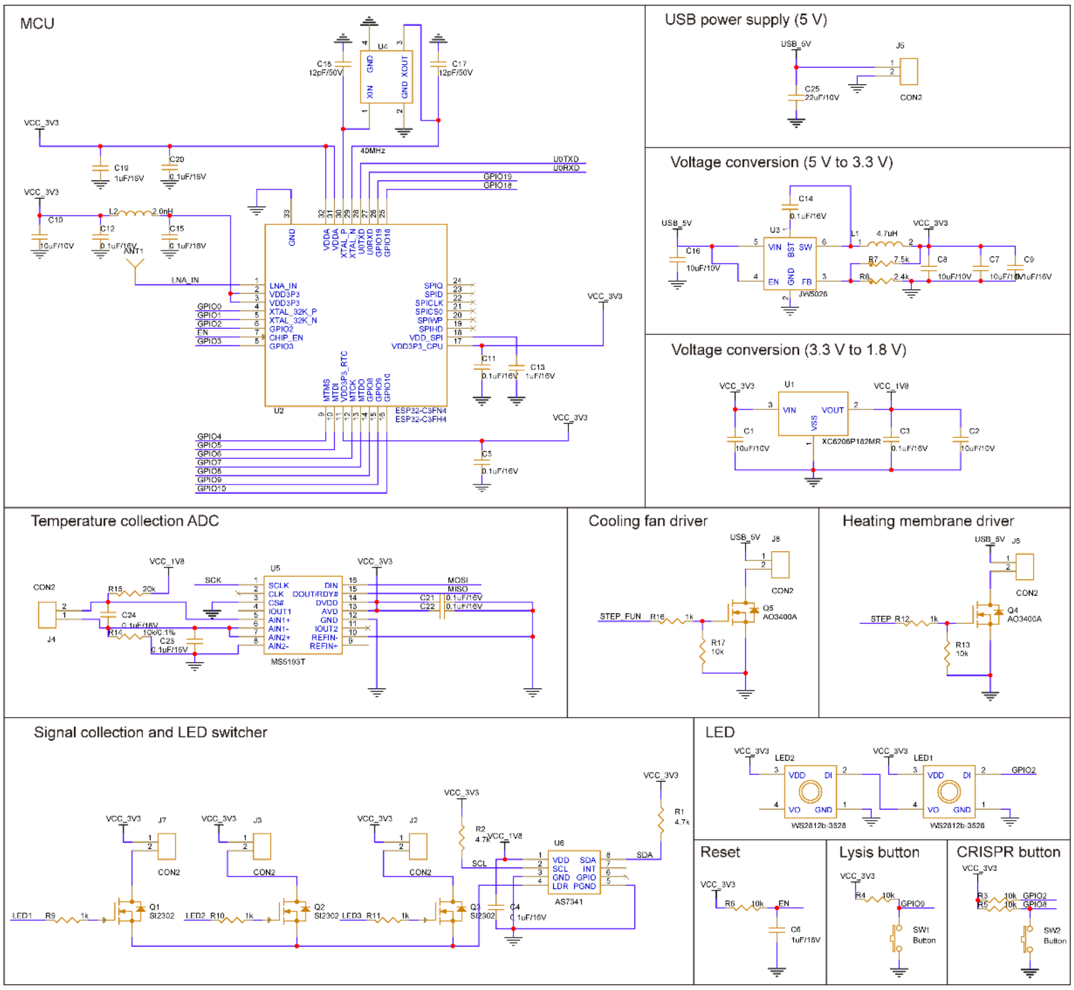

Supplementary Fig. 3. The circuit diagrams of CPod.

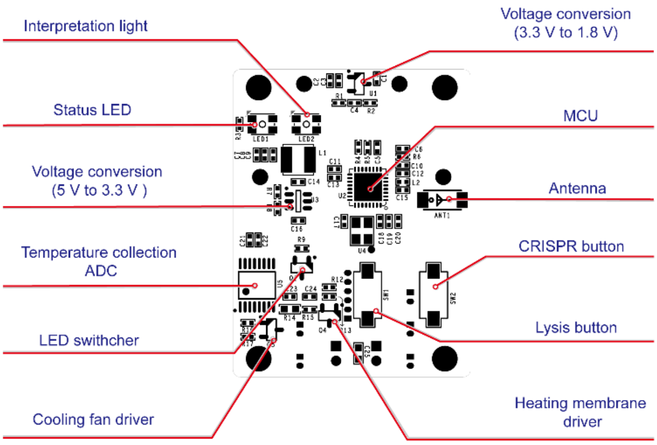

Supplementary Fig. 4. The printed circuit board of CPod.

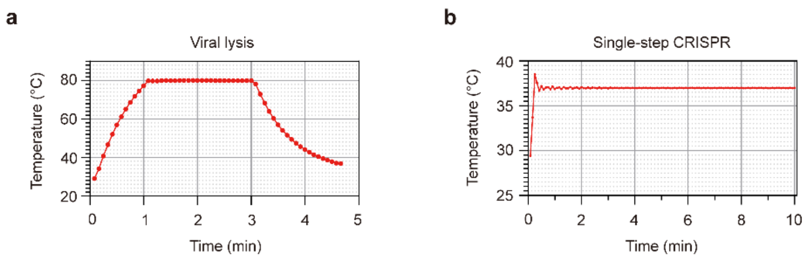

Supplementary Fig. 5. Thermal control performance of CPod. The real-time temperature curves of (**a**) viral lysis and (**b**) single-step CRISPR. The temperature was collected per 5s.

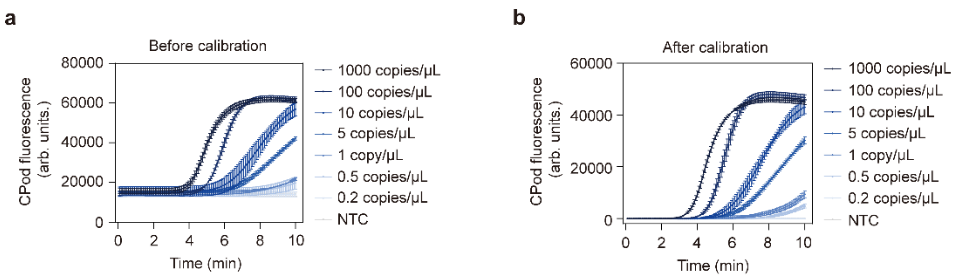

Supplementary Fig. 6. Comparison of the real-time curves tested by CPod before and after calibration. The real-time fluorescence curve of single-step RPA-CRISPR/Cas13a reaction tested by CPod with a gradient concentration of monkeypox virus F3L gene DNA (1000, 100, 10, 5, 1, 0.5, 0.2 copies/μL and NTC) (**a**) before calibration and (**b**) after calibration. Error bars represent the standard deviation of three repetitive experiments. Mean ± s.d. for 3 technical replicates for **a** and **b**.

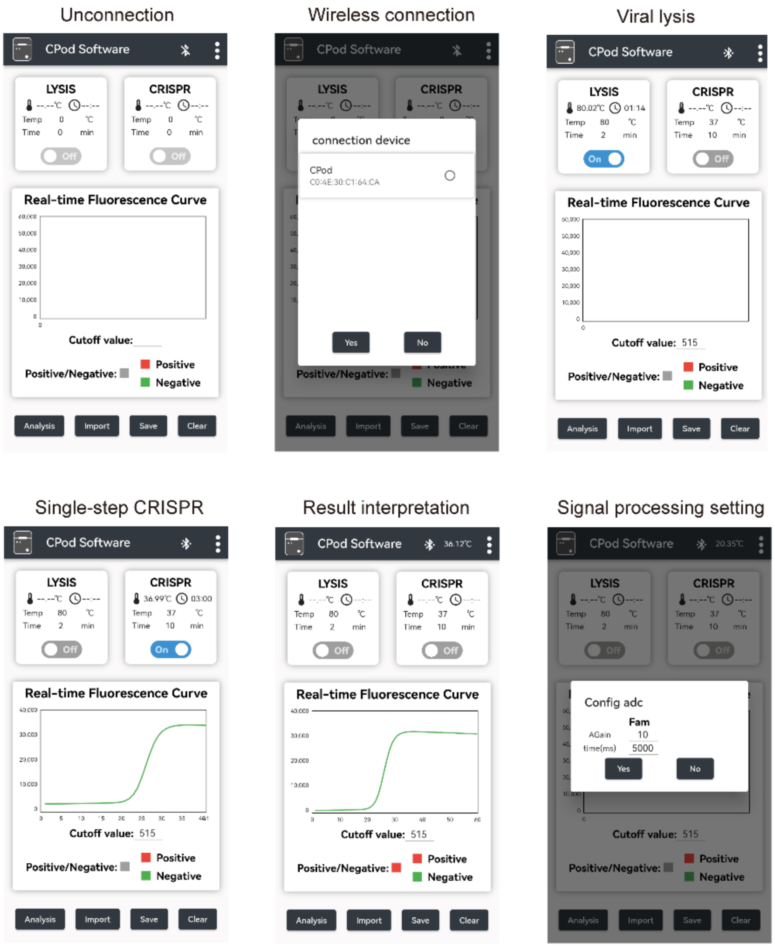

Supplementary Fig. 7. The smartphone software interface for the wireless control of CPod.

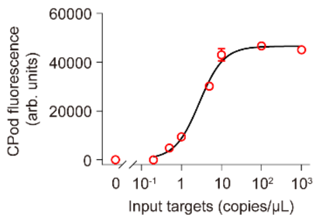

$Y = Bottom + \frac{Top - Bottom}{1+10^{(logEC50 - x)*HillSlope}}$	$R^2 = 0.9889$
Top = 46557	*Bottom* = 151.3
EC50 = 2.803	*HillSlope* = 1.436

Supplementary Fig. 8. The nonlinear regression fitting for the sensitivity testing results of CPod. Mean ± s.d. for 3 technical replicates.

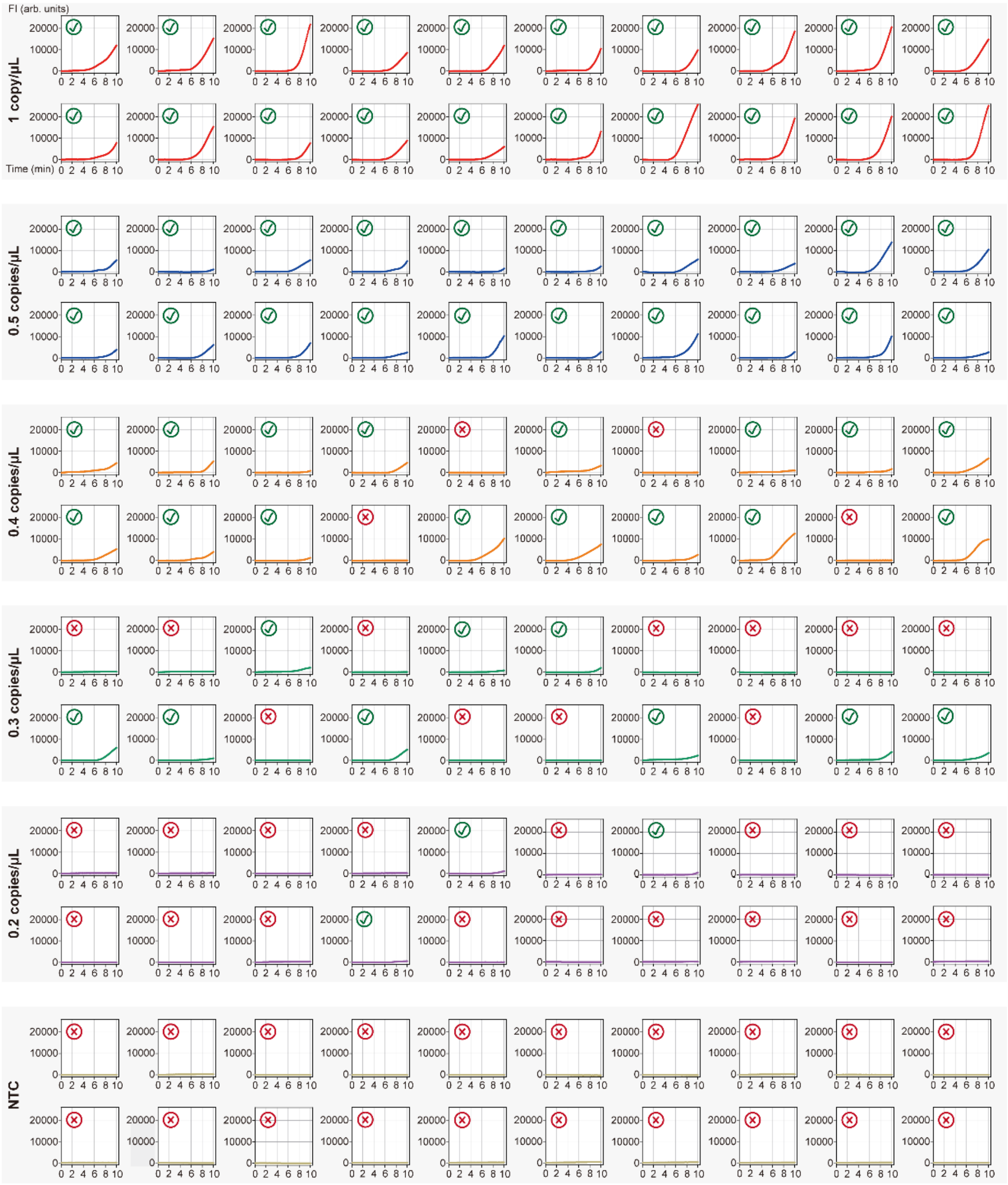

Supplementary Fig. 9. Detection performance with the low concentration of targets. The real-time fluorescence intensity (FI) curve of single-step RPA-CRISPR/Cas13 reaction tested by CPod using the target with the concentration of 1, 0.5, 0.4, 0.3, 0.2 copies/μL, and NTC, independently repeated 20 times.

a

qPCR assay

Sample number	1	2	3	4	5	6	7	8	9	10	11	12	13	14	15	16	17	18	19	20	21	22	23	24
Ct value	*27.97*	*28.22*	*27.86*	*28.03*	*33.37*	*33.38*	*33.50*	*34.34*	*36.50*	*37.07*	*36.70*	*36.38*	*37.85*	*37.97*	*38.79*	*38.76*	*41.86*	*41.52*	*41.71*	*41.53*	*n.d.*	*n.d.*	*n.d.*	*n.d.*

b

CRISPR assay

Fluorescence (arb. units)
0 10000 20000 30000 40000

Sample number	1	2	3	4	5	6	7	8	9	10	11	12	13	14	15	16	17	18	19	20	21	22	23	24
Fluorescence	+	+	+	+	+	+	+	+	+	+	+	+	+	+	+	+	+	+	+	+	-	-	-	-

c

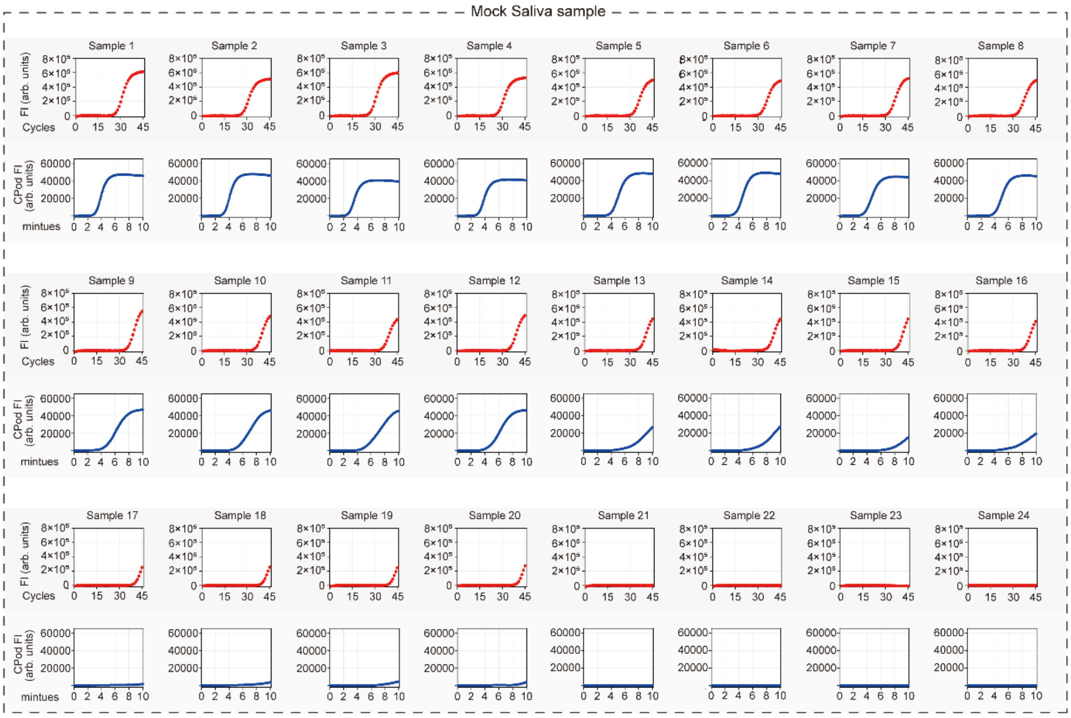

Supplementary Fig. 10. Validation of the clinical performance of SCOPE via mock saliva samples. a, The Ct values of 24 mock clinical samples tested by qPCR. ‘n.d.’ means not detected. **b**, The corresponding fluorescent intensities at 10 min for 48 mock clinical samples tested by our CRISPR-based assay. ‘+’ means a positive test, ‘-’ means a negative test. **c**, The real-time fluorescence curves of qPCR tested by plate reader (red) and single-step RPA-CRISPR/Cas13 tested by CPod (blue) with mock saliva samples. FI means fluorescence intensity.

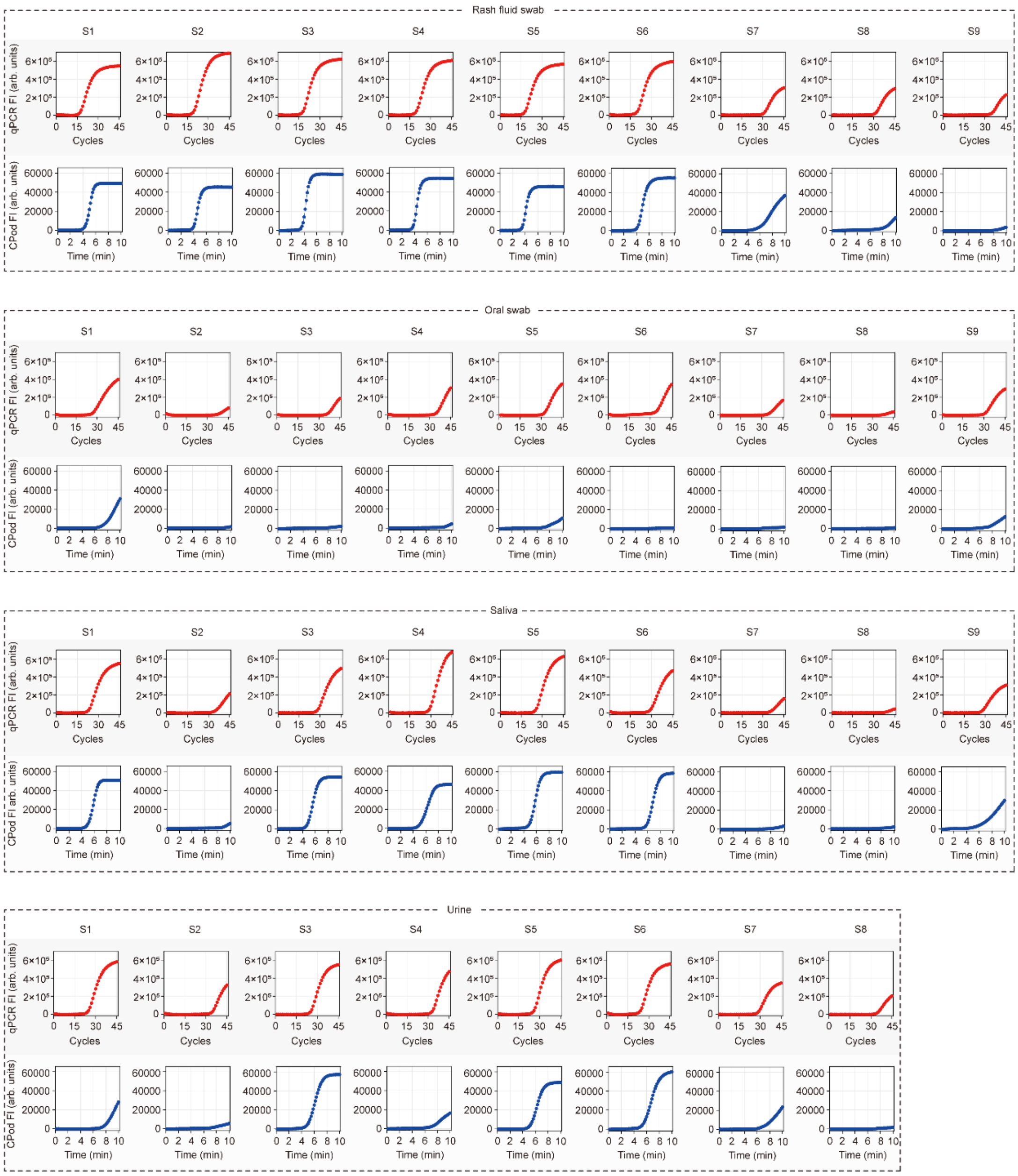

Supplementary Fig. 11. Validation of the clinical performance of our CRISPR-based detection system via 35 monkeypox virus positive samples. The real-time fluorescence curve of qPCR (red) tested by plate reader and single-step RPA-CRISPR/Cas13 (blue) tested by CPod with monkeypox virus positive rash fluid swab, oral swab, saliva, and urine samples. FI means fluorescence intensity.

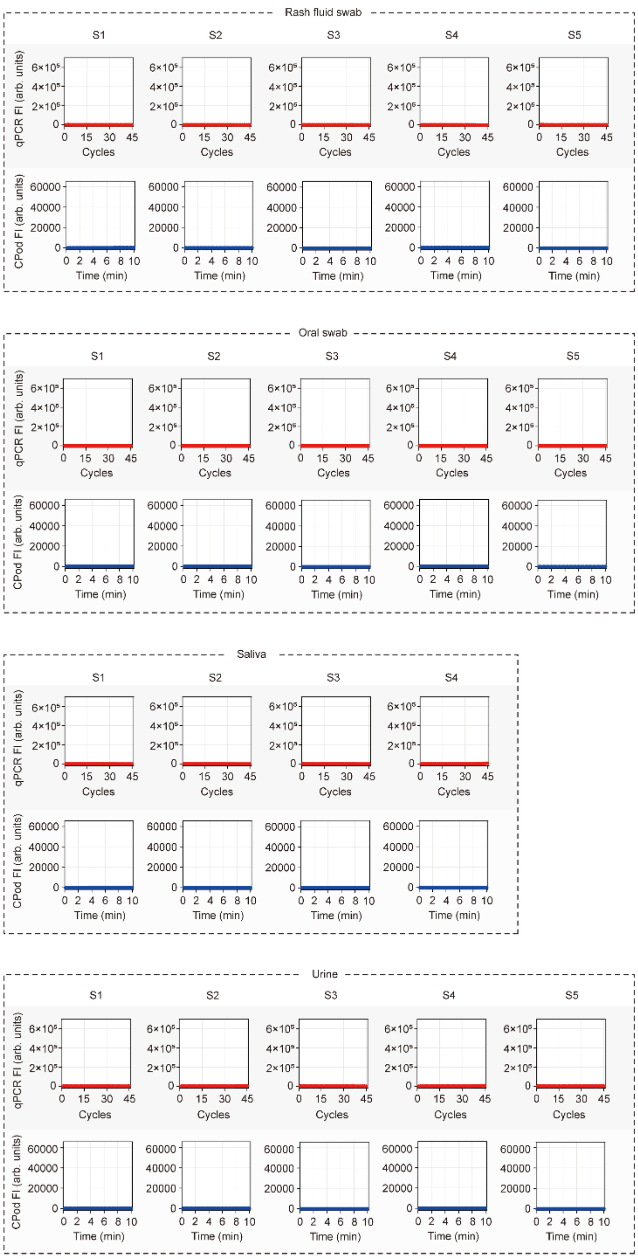

Supplementary Fig. 12. Validation of the clinical performance of our CRISPR-based detection system via 19 herpes simplex virus samples. The real-time fluorescence curve of qPCR (red) tested by plate reader and single-step RPA-CRISPR/Cas13 (blue) tested by CPod with herpes simplex virus positive rash fluid swab, oral swab, saliva, and urine samples. FI means fluorescence intensity.

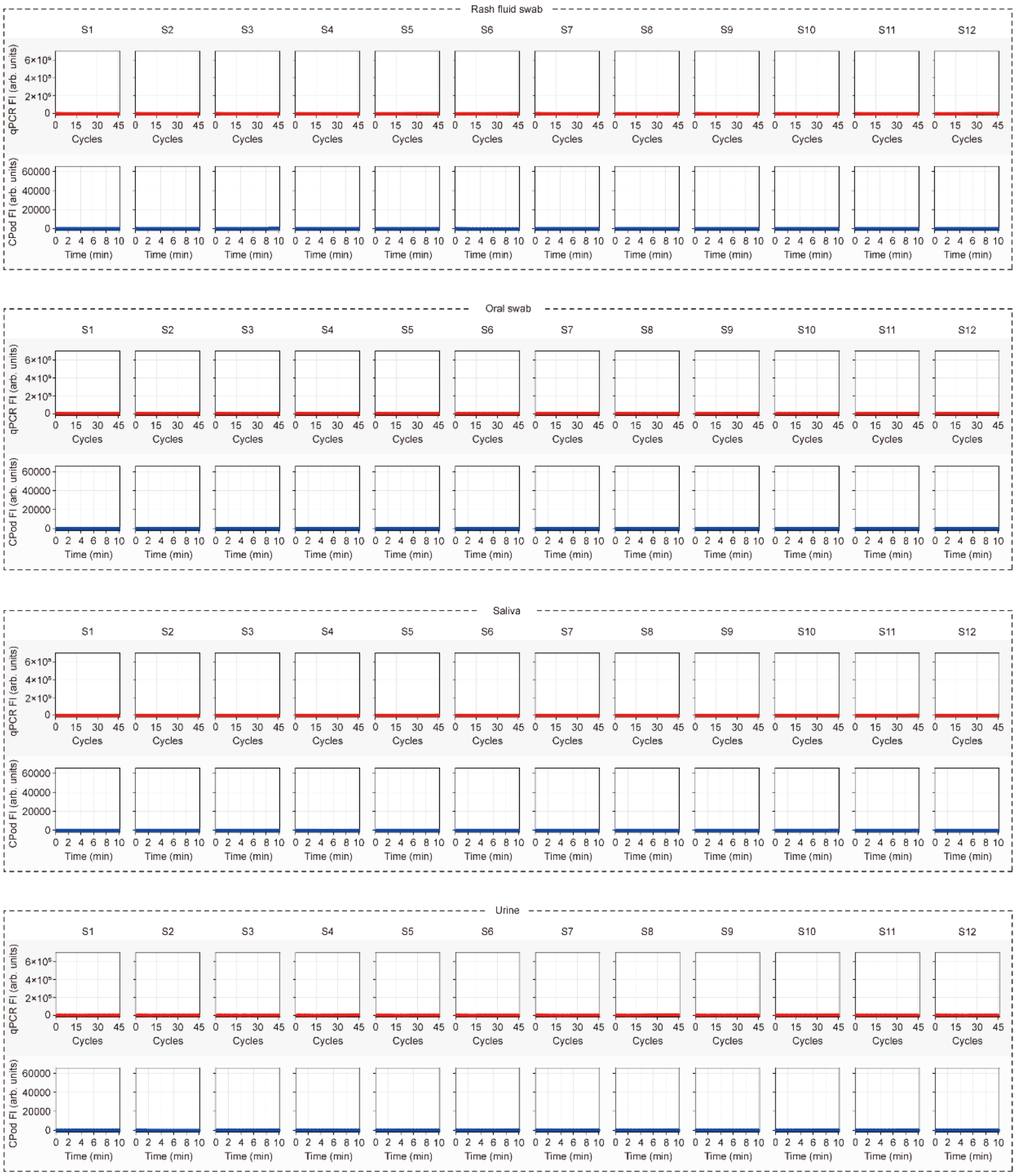

Supplementary Fig. 13. Validation of the clinical performance of our CRISPR-based detection system via 48 negative samples. The real-time fluorescence curve of qPCR (red) tested by plate reader and single-step RPA-CRISPR/Cas13 (blue) tested by CPod with monkeypox virus negative rash fluid swab, oral swab, saliva, and urine samples. FI means fluorescence intensity.

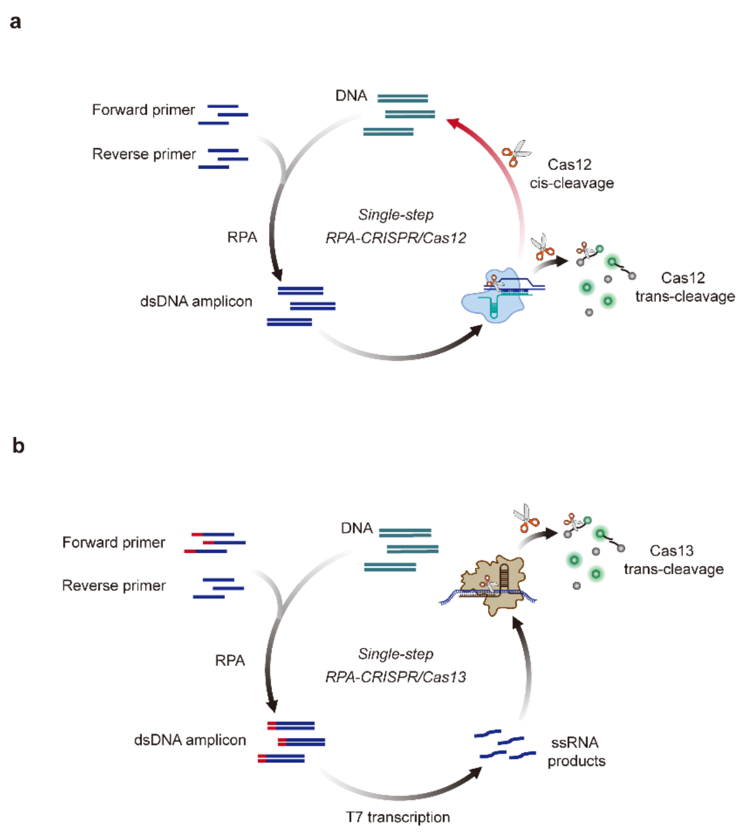

Supplementary Fig. 14. The detection mechanisms of (**a**) single-step RPA/CRISPR/Cas12 and (**b**) single-step RPA/CRISPR/Cas13.

Supplementary Table 1. The list of the CPod components.

Components	Item	Catalog	Manufacturer	Quantity	Price ($)	Cost ($)
Electronical components	MCU	ESP32-C3FN4	ESPRESSIF	1	0.84	0.84
	Antenna	AN2051-245	Rainsun	1	0.1498	0.1498
	Analog to Digital Converter	MS5193T	HangZhou Relmon	1	3.08	3.08
	Crystal	XC32M4-40.000-F12NJDT	Hangjing	1	0.056	0.056
	Temperature transducer	Mf54k	NanJing Sino Chip	1	0.539	0.539
	Cooling fan	30*30*7 mm, 5V, 11000 rpm	ShenZhen QISU	1	1.03	1.03
	Heating membrane	5V, 5W	Custom-made	1	0.7	0.7
	470nm LED	C503B-BAN-CZ0A0452	Wolfspeed	1	0.196	0.196
	Optical sensor	AS7341	AMS OSRAM	1	1.86	1.86
	DC-to-DC Converter	JW5026	Joulwatt	1	0.098	0.098
	Precision resistance	10k/0.1%,	VISHAY	1	0.028	0.028
	Button	TS-1101-C-W	XKB	2	0.0154	0.0308
	Low dropout regulator	XC6206P182MR	UMW	1	0.056	0.056
	Resistance	/	YAGEO	21	0.0014	0.0294
	Field effect transistor	SI2302DS	JSMSEMI	1	0.014	0.014
		AO3400A		2	0.014	0.028
	Inductor	2.0nH	muRata	1	0.014	0.014
		4.7uH		1	0.014	0.014
	LED	RGB	MEIHU	2	0.0308	0.0616
	Connector	1.25-3A	DEALON	1	0.0056	0.0056
		1.25-2A		2	0.0056	0.0112
		1.25-6A		1	0.0112	0.0112
		ph200mm-1x2	HOOYA	1	0.014	0.014
		ph200mm-1x4	HOOYA	2	0.021	0.042
		1.25mm 1x6P	HongCheng	1	0.0224	0.0224
	Capacitance	12pF/50V	muRata	2	0.0007	0.0014
		10uF		10	0.0042	0.042
		22uF/10V		3	0.0098	0.0294

		1uF/16V		3	0.0462	0.1386
		0.1uF/16V		14	0.0014	0.0196
	USB connector	TYPE-C-31-M-12	HRO	1	0.112	0.112
	Demo board	USB board	Shenzhen JLC	1	0.021	0.021
		Transducer board	Shenzhen JLC	1	0.0182	0.0182
	Mainboard	/	Shenzhen JLC	1	0.084	0.084
	Line	/	/	1	0.07	0.07
	Welding	/	/	1	0.56	0.56
Mechanical componets	Shell	/	Custom-made	1	3.28	3.28
	Metal components	/	Custom-made	1	0.07	0.07
	Magnet	/	/	2	0.035	0.07
	Screw	/	/	/	0.014	0.014
	**Total**	/	/	/	/	13.461

Supplementary Table 2. Comparison of our developed assay with the existing CRISPR-Dx assay.

Item	Name	Assay	Detection unit	Sensitivity	Time<br>R: Reaction time<br>W: Whole time	lyophilization	Extraction-free	Reference
**Amplification-free assay**	/	CRISPR/Cas13a	Homemade device	100 copies/μL	30 min (R)	×	×	(*32*)
	/	CRISPR/Cas13a	Droplet chip	Single-molecule	1 h (R)	×	×	(*19*)
	/	CRISPR/Cas13a	Field-effect transistors	1 aM	30 min	×	√	(*16*)
	CONAN	Cas12 cascade	Plate reader	3 copies/μL	30 min	×	×	(*17*)
	MOPCS	CRISPR/Cas12	Surface plasmon resonance	15 fM	38 min	×	×	(*33*)
**Two-step assay**	DETECTR	RT-LAMP, Cas12a	LFA	10 copies/μL	45 min (R)	×	×	(*22*)
	ADESSO	RT-RPA, Cas13a	Plate reader or LFA	Ct ~30	1 h (W)	×	√	(*34*)
	DISCoVER	RT-LAMP, Cas13a	Microfluidic chip	40 copies/μL	1 h (W)	×	√	(*35*)
	RAY	RT-PCR, Cas9	LFA	Ct 34	55 min (R)	×	×	(*36*)
	HOLMES	PCR, Cas12a	Plate reader	1 aM	1 h (R)	×	×	(*23*)
	MAPnavi	RT-RPA, Cas12a	Microfluidic chip	200 copies/mL	< 40 min (W)	√	√	(*37*)
	/	RT-RPA, Cas12a	LFA on the mask	500 copies/μL	90 min (W)	√	√	(*38*)
	DAMPR	RT-LAMP, Cas9	Homemade device	100 copies/μL	50 min (R)	×	×	(*39*)
	MiCaR	RPA, Cas12a	Microfluidic chip	0.26 aM	40 min (R)	×	√	(*40*)
**Single-step assay**	miSHERLOCK	RT-RPA, Cas12a	Homemade device	1100 copies/mL	1 h (W)	√	√	(*41*)
	HOLMESv2	LAMP-Cas12b	Plate reader	10^{-8} nM	120 min (R)	×	×	(*42*)
	/	RT-RPA-Cas12a	Homemade device	0.38 copies/μL	15 min (W)	×	√	(*43*)
	SHINE	RT-RPA-Cas13a	Plate reader or LFA	100 copies/μL	55 min (W)	√	√	(*25*)
	AIDO-CRISPR	RT-RPA-Cas12a	Portable LED and ImageJ	5 copies/μL	20 min (R)	×	×	(*24*)
	STOPCovid	RT-LAMP-Cas12b	Plate reader or LFA	Ct 40.3	1 h (W)	×	√	(*44*)
	CRISPR-SPADE	RT-LAMP-Cas12b	Homemade device	12 copies/μL	20-30 min (R)	√	×	(*45*)
	sPAMC	RT-RPA-Cas12a	Plate reader	Ct ~35.8	~20 min	×	√	(*46*)
	SHINEv2	RT-RPA-Cas13a	Plate reader or LFA	200 copies/μL	< 90 min (W)	√	√	(*27*)
	SCOPE	RPA-Cas13a	Homemade tiny device	0.5 copies/μL	15 min (W)	√	√	This work

Supplementary Table 3. Comparison of our developed assay with the existing MPXV detection assay.

Assay	Detection unit	Sensitivity	Time R: Reaction time W: Whole time	Clinical verification	lyophilization	Extraction-free	Reference
Amplification-free CRISPR/Cas12a	Surface plasmon resonance-based fiber tip biosensor	59.5 copies/μL	1.5 h (R)	×	×	×	(47)
Single-step RPA-CRISPR/Cas12a	Plate reader or visual detection	15 copies/μL	35 min (W)	×	×	×	(48)
LAMP	End-point detection or plate reader	28.7 copies/reaction	30 min (R)	×	×	√	(49)
Single-step RPA-CRISPR/Cas12a	UV light	10 virus particles	25 min (W)	×	×	√	(50)
Single-step RPA-CRISPR/Cas12a	Plate reader	1 copy/μL	30 min (R)	1 Mpox Case	×	×	(51)
DNAzyme-based chemiluminescence CRISPR/Cas12a	Smartphone-assisted imaging cartridge	5.2 copy/μL	> 1 h (R)	×	×	×	(52)
RPA, CRISPR/Cas12a	Plate reader or LFA	1 copy/μL	45 min (R)	40 Mpox cases	×	×	(53)
RPA, CRISPR/Cas12a, nanopore	Glass nanopore electronic sensor	16 copy/μL	55 min (R)	×	×	×	(54)
Single-step RPA-CRISPR/Cas12a	Portable device	10.6 particles/μL	35 min (W)	×	√	√	(55)
RAA, Cas12a/Cas13a	Plate reader	4 copies/reaction	< 40 min (R)	×	×	×	(56)
Single-step RPA-CRISPR/Cas13a	Homemade tiny device	0.5 copies/μL	15 min (W)	35 Mpox cases	√	√	This work

Supplementary Table 4. Clinical sample information.

Sample	ID	Gender	Age	Sample type	Viral Ct
Monkeypox virus positive	S1	Male	28	Rash fluid swab	15.88
				Oral swab	25.38
				Saliva	21.53
				Urine	22.75
	S2	Male	28	Rash fluid swab	18.49
				Oral swab	39.02
				Saliva	34.51
				Urine	34.06
	S3	Male	33	Rash fluid swab	16.84
				Oral swab	36.54
				Saliva	27.59
				Urine	24.62
	S4	Male	30	Rash fluid swab	18.64
				Oral swab	34.56
				Saliva	27.95
				Urine	32.19
	S5	Male	30	Rash fluid swab	16.84
				Oral swab	31.99
				Saliva	24.62
				Urine	24.86
	S6	Male	26	Rash fluid swab	19.15
				Oral swab	32.27
				Saliva	28.67
				Urine	23.18
	S7	Male	37	Rash fluid swab	28.97
				Oral swab	33.23
				Saliva	33.83
				Urine	26.68
	S8	Male	28	Rash fluid swab	29.69
				Oral swab	38.28
				Saliva	38.11
				Urine	33.31
	S9	Male	31	Rash fluid swab	32.62
				Oral swab	28.9
				Saliva	26.06
Herpes simplex virus positive	S1	Male	68	Rash fluid swab	-
				Oral swab	-
				Saliva	-
				Urine	-
	S2	Male	66	Rash fluid swab	-
				Oral swab	-

				Saliva	-
				Urine	-
	S3	Male	69	Rash fluid swab	-
				Oral swab	-
				Saliva	-
				Urine	-
	S4	Male	40	Rash fluid swab	-
				Oral swab	-
				Saliva	-
				Urine	-
	S5	Male	60	Rash fluid swab	-
				Oral swab	-
				Urine	-
Healthy control	S1	Male	36	Rash fluid swab	-
				Oral swab	-
				Saliva	-
				Urine	-
	S2	Male	37	Rash fluid swab	-
				Oral swab	-
				Saliva	-
				Urine	-
	S3	Male	27	Rash fluid swab	-
				Oral swab	-
				Saliva	-
				Urine	-
	S4	Male	35	Rash fluid swab	-
				Oral swab	-
				Saliva	-
				Urine	-
	S5	Male	26	Rash fluid swab	-
				Oral swab	-
				Saliva	-
				Urine	-
	S6	Male	33	Rash fluid swab	-
				Oral swab	-
				Saliva	-
				Urine	-
	S7	Male	32	Rash fluid swab	-
				Oral swab	-
				Saliva	-
				Urine	-
	S8	Male	24	Rash fluid swab	-
				Oral swab	-

				Saliva	-
				Urine	-
	S9	Male	25	Rash fluid swab	-
				Oral swab	-
				Saliva	-
				Urine	-
	S10	Male	26	Rash fluid swab	-
				Oral swab	-
				Saliva	-
				Urine	-
	S11	Male	28	Rash fluid swab	-
				Oral swab	-
				Saliva	-
				Urine	-
	S12	Male	28	Rash fluid swab	-
				Oral swab	-
				Saliva	-
				Urine	-

Supplementary Table 5. The information of material used in this study.

Reagent	Reaction	Manufacturer	Cat.no.	Stock Concentration
LwaCas13a	Single-step CRISPR	Tolo Biotech Co., Ltd	32117-01	10 μM
RPA lyophilized pellets	Single-step CRISPR	TwistDx™	TABAS03KIT	/
Rehydration buffer	Single-step CRISPR	TwistDx™	TABAS03KIT	/
T7 RNA polymerase	Single-step CRISPR	New England Biolabs® Inc.	M0251	50 U/μL
RNase H	Single-step CRISPR	New England Biolabs® Inc.	M0297	5 U/μL
RNase Inhibitor	Single-step CRISPR	Solarbio® Life Science	R8061	40 U/μL
rNTP mix	Single-step CRISPR	New England Biolabs® Inc.	N0466	25 mM
Monkeypox pseudovirus	Single-step CRISPR and PCR	Fubio	FNDV4731	/
DNase/RNase-free water	/	Solarbio® Life Science	R1600	/
HiScribe ™ T7 High Yield RNA Synthesis Kit	IVT	New England Biolabs® Inc.	E2040	/
Standard Taq buffer	IVT	New England Biolabs® Inc.	B9014	10×
RNA Clean & Concentrator-5 Kit	IVT	ZYMO RESEARCH	R1013	/
RQ1 RNase-Free DNase	IVT	Promega	M6101	10 U/μL
Qubit™ RNA Broad Range Assay Kits	IVT	Thermo Fisher Scientfic™	Q10211	/
Viral Extraction kit	Viral extraction	Tiangen Biotech	DP315-R	/
Chelex-100	Viral lysis	Solarbio® Life Science	C8230	/
TCEP	Viral lysis	Beyotime Biotechnolog y	ST049	500 mM

Tris (pH8.0)	Viral lysis	Thermo Fisher Scientfic^{TM}	15568025	1 M
BSA	Lyophilization	Sigma-Aldrich	10711454001	/
Taq buffer	qPCR	Fapon Biotech	MD099M	5×
Taq master mix	qPCR	Fapon Biotech	MD099M	25×
Loading dye	Agarose gel electrophoresis	NEB	B7021S	6×
Agarose	Agarose gel electrophoresis	Invitrogen	75510019	/
DNA marker	Agarose gel electrophoresis	Tiangen	MD108	/
Disposable virus sampling tube	Sample collection	Yocon Biology	MT0301	/
Disposable sterile sampling cup	Sample collection	BKMAM Biotechnology	110413005	/

Supplementary Table 6. The sequences of primers and CRISPR crRNA used in this study.

Name	***Sequence (5' - 3')***
MPXV-RPA-F1	*cctctaatacgactcactataggg*TTGATTTTTCGCGGGATACATCATCTATTA
MPXV -RPA-F2	*cctctaatacgactcactataggg*CTCATTGATTTTTCGCGGGATACATCATCT
MPXV -RPA-F3	*cctctaatacgactcactataggg*TCCTCTCTCATTGATTTTTCGCGGGATACA
MPXV -RPA-F4	*cctctaatacgactcactataggg*TCAAAAGACTTATGATCCTCTCTCATTGAT
MPXV -RPA-R1	AACGATACTCCTCCTCGTTGGTCTACGACA
MPXV-RPA-R2	ATGGTTTACAGCTCCAACGATACTCCTCCT
MPXV-RPA-R3	GTAGTGCTATGGTTTACAGCTCCAACGATA
MPXV-RPA-R4	ATGATCTTCAACGTAGTGCTATGGTTTACA
MPXV-crRNA1	ATCAGAATCTGTAGGCCGTGTATCAGCA*gttttagtccccttcgtttttggggtagtctaaatcccctatagtgagtcgtattaatttc*
MPXV-crRNA2	AGCATCAGCATCAGAATCTGTAGGCCGT*gttttagtccccttcgtttttggggtagtctaaatcccctatagtgagtcgtattaatttc*
MPXV-crRNA3	GAATCTGTAGGCCGTGTATCAGCATCCA*gttttagtccccttcgtttttggggtagtctaaatcccctatagtgagtcgtattaatttc*
MPXV-PCR-F	GCATTATTTTTAGCATCTCGT
MPXV-PCR-R	TTATGCCTGTGTAGACATTGAC
MPXV-PCR-P	FAM-TCCATCTGCCTTATCGAATACTCT-BHQ1
T7-3G-IVT primer	GAAATTAATACGACTCACTATAGGG

Supplementary Table 7. The sequences of synthesized MPXV, Cowpox, Vaccinia, and Variola pseudovirus F3L gene.

**Virus**	**Sequence (5’ - 3’)**
Monkeypox	TCAGAATCTAATGATGACATAACTAAGAAGTTTATCTACAGCCAATTTAG<br>CTGCATTATTTTTAGCATCTCGTTTAGATTTTCCATCTGCCTTATCGAATA<br>CTCTTCCGTCAATGTCTACACAGGCATAAAATGTAGGAGAGTTACTAGG<br>CCCCACTGATTCAATACGAAAAGACCAATCTCTCCTAGTTATTTGACAG<br>TACTCATTAATAACGGTGACAGGGTTAACACCTTTCCAATAAATAATTTT<br>TTTAACCGGAATAACATCATCAAAAGACTTATGATCCTCTCTCATTGATT<br>TTTCGCGGGATACATCATCTATTATAGCATCAGCATCAGAATCTGTAGGC<br>CGTGTATCAGCATCCATTGTCGTAGACCAACGAGGAGGAGTATCGTTGG<br>AGCTGTAAACCATAGCACTACGTTGAAGATCATACAGAGCTTTATTAAC<br>TTCTCGCTTCTCCAT
Cowpox	TCAGAATCTAATGATGACGTACCCAAGAAGTTTATCTACAGCCAATTTA<br>GCTGCATTATTTTTAGCATCTCGTTTAGATTTTCCATCGGCCTTATCGAAT<br>ACTCTTCCATCGATGTCTACACAGGCATAAAATGTAGGAGAGTTACTAG<br>GTCCCACTGATTCAATACGAAAAGACCAATCTCTCTTAGTTATTTGGCA<br>GTACTCATTAATAATGGTGACAGGGTTAGCATCTTTCCAATCAATAATTT<br>TTTTGGCAGGAATAACATCATCAAAAGACTTATGATCCTCTCTCATTGAT<br>TTTTCGCGGGATACATCATCTATTATGACGTCAGCCATAACATCAGCATC<br>CGTCTTATCCGCCTCCGTTGTCATAAACCAACGAGGAGGAATATCGTCG<br>GAGCTGTACACCATATCACTACGTTGAAGATCGTACAGAGCTTTATTAA<br>CTTCTCGCTTCTCCATATTAAGTTGTCTAGTTAGTTGTGCAGCAGTAGCT<br>CCTTCGATTCCAATGGTTTTAATAGCCTCACACACAATCTCTGCGTCAG<br>AACGTTCGTCGATATAGATTTTAGACAT
Vaccinia	TTATTTACCATCCCATATATTCCATGAATAAGTGTGATGATTGTACACTTC<br>TATAGTATCTATATACGATTCACGATAAAATCCTCCTATCAATAGCAGTTT<br>ATTATCCACTATGATCAATTCTGGATTATCCCTCGGATAAATAGGATCATC<br>TATCAGAGTCCATGTATTGCTGGATTCACAATAAAATTCCGCATTTCTAC<br>CAACCAAGAATAACCTTCTACCGAACACTAACGCGCATGATTTATAATG<br>AGGATAATAAGTGGATGGTCCAAACTGCCACTGATCATGATTGGGTAGC<br>AAATATTCTGTAGTTGTATCAGTTTCAGAATGTCCTCCCATTACGTATATA<br>ACATTGTTTATAGATGCCACTGCTGGATTACATCTAGGTTTCAGAAGACT<br>CGGCATATTAACCCAAGCAGCATCCCCGTGGAACCAACGCTCAACAGA<br>TGTGGGATTTGGTAGACCTCCTACTACGTATAATTTATTGTTAGCGGGTA<br>TCCCGCTAGCATACAGTCTGGGGCTATTCATCGGAGGAATTGGAATCCA<br>ATTGTTTGATATATAATTTACAGCTATAGCATTGTTATGTATTTCATTGTTC<br>ATCCATCCACCGATGAGATATACTACTTCTCCAACATGAGTACTTGTACA<br>CATATGGAATATATCTATAATTTGATCCATGTTCATAGGATACTCTATGAAT<br>GGATACTTGTATGATTTGCGTGGTTGTTTATCACAATGAAATATTTTGGT<br>ACAGTCTAGTATCCATTTTACATTATTTATACCTCTGGGAGAAAGATAATT<br>TGACCTGATTACATTTTTGATAAGGAGTAGCAGATTTCCTAATTTATTTCT<br>TCGCTTTATATACCACTTAATGACAAAATCAACTACATAATCCTCATCTG<br>GAACATTTAGTTCATCGCTTTCTAGAATAAGTTTCATAGATAGATAATCA

	AAATTGTCTATGATGTCATCTTCCAGTTCCAAAAAGTGTTTGGCAATAAAGTTTTTAGTATGACATAAGAGATTGGATAGTCCGTATTCTATACCCATCATGTAACACTCGACACAATATTCCTTTCTAAAATCTCGTAAGATAAAGTTTATACAAGTGTAGATGATAAATTCTACAGAGGTTAATATAGAAGCACGTAATAAATTGACGACGTTATGACTATCTATATATACCTTTCCAGTATATGAGTAAATAACTATAGAAGTTAAACTGTGAATGTCAAGGTCTAGACAAACCCTTGTAACTGGATCTTTATTTTTCGTGTATTTTTGACGTAAATGTGTGCGAAAGTAAGGAGATAACTTTTTCAATATCGTAGAATTGACTATTATATTGCCACCTATAGCATCAATAATTGTTTTGAATTTCTTAGTCATAGACAATGCTAATATATTCTTACAGTACACAGTATTAACAAATATCGGCAT
Variola	TCAATTCTGGATTATCCCTTGATAAATAGTATCATCTATCAGAGACCATGTATTGCTGTATTTGTAATAAAATTTAGCATTTCTACCAACCAAGAATAACCTTCTACCGAACACTAACGCGCATGATTTATAATGAGGATAATAAGTTGACGGTCCAAACTGCCACTGATCATGATTGGGTAGCAAATATTCTGTAGTTGTATCCGTTTCAGAATGTCCTCCTATTACGTACATAACATTGTTTATGGATGCCATTGCTGGATTACATCTAGGTTTCAAAAGACTTGGCATATTAACCCAAGCAGCATTCCCGTGGAACCAACGCTCAACAGATGTGGGATTTGGTAGACCTCCTACTACGTATAATTTATTGTTAGCGGGTATCCCGCTAGCATACAGTTTGGGGCTATTCATCGGAGGAATTGGAATCCAATTGTTTGATATATAATTTACCGCTATAGCATTGTTATGTATTTCATTGTTCAT
