# Supplementary material for: Ultrasensitive single-step CRISPR detection of monkeypox virus in minutes with a vest-pocket diagnostic device
Source: Nat Commun. 2024 Apr 16;15:3279. doi: 10.1038/s41467-024-47518-8 (PMC11021474; doi:10.1038/s41467-024-47518-8)
Supplement: Supplementary file 2 — Description of additional supplementary files [file 41467_2024_47518_MOESM2_ESM.pdf]

Description of Additional Supplementary Files

Supplementary Movie 1: Standalone mode of SCOPE for monkeypox virus detection.

Supplementary Movie 2: Wireless control mode of SCOPE for monkeypox virus detection.
