# Supplementary material for: Ultrasensitive single-step CRISPR detection of monkeypox virus in minutes with a vest-pocket diagnostic device
Source: Nat Commun. 2024 Apr 16;15:3279. doi: 10.1038/s41467-024-47518-8 (PMC11021474; doi:10.1038/s41467-024-47518-8)
Supplement: Supplementary file 6 — Reporting Summary [file 41467_2024_47518_MOESM6_ESM.pdf]

nature portfolio

Corresponding author(s): Zhen Rong, Shengqi Wang

Last updated by author(s): Mar 14, 2024

Reporting Summary

Nature Portfolio wishes to improve the reproducibility of the work that we publish. This form provides structure for consistency and transparency in reporting. For further information on Nature Portfolio policies, see our Editorial Policies and the Editorial Policy Checklist.

Statistics

For all statistical analyses, confirm that the following items are present in the figure legend, table legend, main text, or Methods section.

n/a	Confirmed	
☐	☒	The exact sample size (n) for each experimental group/condition, given as a discrete number and unit of measurement
☐	☒	A statement on whether measurements were taken from distinct samples or whether the same sample was measured repeatedly
☐	☒	The statistical test(s) used AND whether they are one- or two-sided<br>*Only common tests should be described solely by name; describe more complex techniques in the Methods section.*
☒	☐	A description of all covariates tested
☒	☐	A description of any assumptions or corrections, such as tests of normality and adjustment for multiple comparisons
☐	☒	A full description of the statistical parameters including central tendency (e.g. means) or other basic estimates (e.g. regression coefficient) AND variation (e.g. standard deviation) or associated estimates of uncertainty (e.g. confidence intervals)
☐	☒	For null hypothesis testing, the test statistic (e.g. F, t, r) with confidence intervals, effect sizes, degrees of freedom and P value noted<br>*Give P values as exact values whenever suitable.*
☒	☐	For Bayesian analysis, information on the choice of priors and Markov chain Monte Carlo settings
☒	☐	For hierarchical and complex designs, identification of the appropriate level for tests and full reporting of outcomes
☒	☐	Estimates of effect sizes (e.g. Cohen's d, Pearson's r), indicating how they were calculated

Our web collection on statistics for biologists contains articles on many of the points above.

Software and code

Policy information about availability of computer code

Data collection	Real time fluorescence tested by plate reader was determined using Applied Biosystems 7500 Real-Time PCR system. The code for smartphone app is available on Github at http://github.com/zayden/cpod/. Gel imaging was captured by using the Tanon 4600 gel imaging system. The structure of homemade device was modeled by Solidworks 2017. The circuits of device were developed by using Cadence 22.1. The smartphone software was designed by using Flutter 3.16.
Data analysis	The data in this study was generated and analyzed via Graphpad Prism 8 except the rader charts in Fig. 3m and Fig. 3r were generated by Origin 2021.

For manuscripts utilizing custom algorithms or software that are central to the research but not yet described in published literature, software must be made available to editors and reviewers. We strongly encourage code deposition in a community repository (e.g. GitHub). See the Nature Portfolio guidelines for submitting code & software for further information.

Data

Policy information about availability of data

All manuscripts must include a data availability statement. This statement should provide the following information, where applicable:

- Accession codes, unique identifiers, or web links for publicly available datasets
- A description of any restrictions on data availability
- For clinical datasets or third party data, please ensure that the statement adheres to our policy

All the original data of this study are available in the Source Data File.

Research involving human participants, their data, or biological material

Policy information about studies with human participants or human data. See also policy information about sex, gender (identity/presentation), and sexual orientation and race, ethnicity and racism.

Reporting on sex and gender	All the 35 monkeypox positive clinical samples in this study were collected from male patients. According to the epidemiological investigation, most infected individuals in the 2022 monkeypox epidemic were male, thus 19 herplex simple virus samples and 48 negative samples from healthy voluteers were also collected from male patients as the negative control.
Reporting on race, ethnicity, or other socially relevant groupings	*Please specify the socially constructed or socially relevant categorization variable(s) used in your manuscript and explain why they were used. Please note that such variables should not be used as proxies for other socially constructed/relevant variables (for example, race or ethnicity should not be used as a proxy for socioeconomic status).* *Provide clear definitions of the relevant terms used, how they were provided (by the participants/respondents, the researchers, or third parties), and the method(s) used to classify people into the different categories (e.g. self-report, census or administrative data, social media data, etc.)* *Please provide details about how you controlled for confounding variables in your analyses.*
Population characteristics	35 monkeypox virus positive samples, 19 herpes simplex virus positive samples, and 48 monkeypox virus negative samples were collected. Detailed population information was provided in Supplementary Table 4.
Recruitment	Patients with monkeypox virus or herpes simplex virus infection. Personally identifiable information was not collected.
Ethics oversight	This study was performed following the guidelines approved by the Ethics Committee of Beijing Ditan Hospital (approval ID: DTEC-YJ2023-001-02).

Note that full information on the approval of the study protocol must also be provided in the manuscript.

Field-specific reporting

Please select the one below that is the best fit for your research. If you are not sure, read the appropriate sections before making your selection.

☒ Life sciences ☐ Behavioural & social sciences ☐ Ecological, evolutionary & environmental sciences

For a reference copy of the document with all sections, see nature.com/documents/nr-reporting-summary-flat.pdf

Life sciences study design

All studies must disclose on these points even when the disclosure is negative.

Sample size	No statistical methods were used to predetermine sample size. Since variation of across replicates was small and in order to determine the mean and standard error, we determined the sample size of 3 replicates for the experiments of assay development, optimization, and evaluationl. 20 replicates was determined for the test of limit of detection since the variation in detection of low concerntration samples was high and we take the positive detection rate greater than 95% (19/20) as the LoD. Because of the sample volume constrain, experiments of clinical samples verification were conducted with single replicate.
Data exclusions	No data exclusions.
Replication	3 replicates were set up for the experiments of assay development, optimization, and evaluation, and all attempts of replications were successful. 20 replicates were set up for the detection of low concerntration samples to determine the LoD. Single replicate was set up for the clinical samples verfication due to the sample volume constrain.
Randomization	Monkeypox virus samples were randomly allocated for detection.
Blinding	Clinical samples were tested by an individual who was blinded to the qPCR results.

Reporting for specific materials, systems and methods

We require information from authors about some types of materials, experimental systems and methods used in many studies. Here, indicate whether each material, system or method listed is relevant to your study. If you are not sure if a list item applies to your research, read the appropriate section before selecting a response.

Materials & experimental systems

n/a	Involved in the study
☒	☐ Antibodies
☒	☐ Eukaryotic cell lines
☒	☐ Palaeontology and archaeology
☒	☐ Animals and other organisms
☒	☐ Clinical data
☒	☐ Dual use research of concern
☒	☐ Plants

Methods

n/a	Involved in the study
☒	☐ ChIP-seq
☒	☐ Flow cytometry
☒	☐ MRI-based neuroimaging

Plants

Seed stocks

Report on the source of all seed stocks or other plant material used. If applicable, state the seed stock centre and catalogue number. If plant specimens were collected from the field, describe the collection location, date and sampling procedures.

Novel plant genotypes

Describe the methods by which all novel plant genotypes were produced. This includes those generated by transgenic approaches, gene editing, chemical/radiation-based mutagenesis and hybridization. For transgenic lines, describe the transformation method, the number of independent lines analyzed and the generation upon which experiments were performed. For gene-edited lines, describe the editor used, the endogenous sequence targeted for editing, the targeting guide RNA sequence (if applicable) and how the editor was applied.

Authentication

Describe any authentication procedures for each seed stock used or novel genotype generated. Describe any experiments used to assess the effect of a mutation and, where applicable, how potential secondary effects (e.g. second site T-DNA insertions, mosiacism, off-target gene editing) were examined.
